# Dipeptidyl Peptidase-4 Inhibitors as a Third-Line Oral Antihyperglycaemic Agent in Patients with Type 2 Diabetes Mellitus: The Impact of Ethnicity

**DOI:** 10.1155/2014/354040

**Published:** 2014-08-10

**Authors:** X. Zhang, B. Brooks, L. Molyneaux, E. Landy, R. Banatwalla, T. Wu, J. Wong, B. Su, D. K. Yue

**Affiliations:** ^1^Department of Endocrinology and Metabolism, The Second Hospital of Dalian Medical University, Dalian, Liaoning 116027, China; ^2^Diabetes Centre, Royal Prince Alfred Hospital, Camperdown, NSW 2050, Australia; ^3^School of Nursing, University of Sydney, Sydney, NSW 2006, Australia; ^4^Discipline of Medicine, University of Sydney, Sydney, NSW 2006, Australia

## Abstract

*Aims*. The aim of this study is to examine the efficacy of adding a dipeptidyl peptidase-4 (DPP-4) inhibitor to patients with type 2 diabetes inadequately controlled by metformin and sulphonylurea combination treatment. The response of Asian and non-Asian patients to this regimen was also examined. *Methods*. The medical and computerized records of 80 patients were examined. These patients had baseline HbA1c levels ranging from 7.0 to 12.5% and had a DPP-4 inhibitor add-on therapy for a minimum period of 12 weeks. The primary endpoint was the change in HbA1c level before and after DPP-4 inhibitor treatment. *Results*. During oral triple therapy, there was a reduction of HbA1c from 8.3% (7.7–8.9) to 7.2% (6.8–7.6) and 26 patients (32.5%) achieved an HbA1c <7%. Poor baseline glycaemic control, lower BMI, and younger age were associated with a better response, but duration of diabetes and gender did not affect outcome. The HbA1c reduction was not different between Asians and non-Asians group [−1.00% (0.6–1.3) vs −0.90% (0.4–1.6)]. *Conclusions*. DPP-4 inhibitor as a third-line add-on therapy can achieve significant glycaemic improvement in patients with type 2 diabetes inadequately controlled on the combination of metformin and sulphonylurea. The improvement in HbA1c was similar between Asian and non-Asian patients.

## 1. Introduction

The most common combination of oral antihyperglycaemic agents used for patients with type 2 diabetes is metformin and sulphonylurea [1, 2]. Despite good initial efficacy, for most patients, this dual therapy is associated with progressive worsening of blood glucose control over time, requiring additional medication [3]. Clinicians and researchers currently debate the question of which additional agent is best in this situation [4, 5].

In clinical studies, DPP-4 inhibitors have been shown to improve HbA1c significantly when administered as monotherapy or as dual therapy in conjunction with metformin or a sulphonylurea [6–11]. However, information on its use as a third agent in triple therapy is relatively scant. Two studies have demonstrated the efficacy of DPP-4 inhibitors when added to the metformin and sulphonylurea combination [12, 13]. Also of interest is a meta-analysis of 55 studies that has suggested that DPP-4 inhibitors may be more effective in Asian patients [14]. As type 2 diabetes is becoming more common in Asia with the progressive increase in affluence of this region, it is important to determine if the better response to DPP-4 inhibitors in Asian patients can be confirmed. Moreover, the magnitude of the difference needs to be elucidated.

The purpose of the present study is, therefore, to examine the efficacy of DPP-4 inhibitors when given as add-on therapy to the combination of metformin and sulphonylurea in patients with type 2 diabetes and suboptimal glycaemic control. Whether Asian patients respond better to triple therapy was also determined.

## 2. Subject and Methods

### 2.1. Selection of Patients

Patients were selected from those who attended the Diabetes Centre of Royal Prince Alfred Hospital, Sydney, Australia. Their clinical and demographic data were extracted from a purpose built diabetes database which included approximately 25,000 patients collected over two decades. A total of 688 patients with type 2 diabetes were found to be treated with DPP-4 inhibitor (Sitagliptin, Linagliptin, Saxagliptin, or Vildagliptin) over the previous 5 years (from 2008 to 2013). Amongst these, 169 patients were on triple oral therapy using sulphonylurea, metformin, and DPP-4 inhibitors. From this group, patients were selected for this analysis if they met the following exclusion and inclusion criteria:

1. added DPP-4 inhibitor at the maximum recommended dosage to the combination of metformin and sulphonylurea treatment (metformin > 1.5 gm/day or > 1.0 gm/day if on XR preparation, or maximum tolerated dose; Diamicron > 160 mg/day, Diamicron MR ≥ 60 mg/day, Amaryl 4 mg/day, or Minidiab 10 mg/day);
2. had no change in the dosage of sulphonylurea or metformin after DPP-4 inhibitor was added;
3. remained on triple therapy for ≥12 weeks;
4. had HbA1c results available (i) within the 12 months prior to and (ii) between 3 to 12 months after addition of DPP-4 inhibitor therapy;
5. were not on insulin or GLP agonist or other diabetes medications.

Eighty-nine patients were excluded from the analysis. Of these, 49 were on DPP-4 inhibitors as the second agent of the triple therapy and 40 had HbA1c measured at a time outside the specified window. The remaining 80 patients were available for analysis. Ethnicity of the individuals was determined by self-report of the patients (Asian group *n* = 41: 36 Chinese and 5 from the Indian Subcontinent; non-Asian group *n* = 39: 21 Anglo-Celtics, 3 Middle Eastern, 1 Indigenous Australians, and 14 Europeans). Permission to record and analyze the computerized data was given by the ethics committee of the hospital.

### 2.2. Statistical Analysis

Numerical data are presented as percentage or mean ± standard deviation or median and interquartile range. The change in HbA1c before and after DPP-4 inhibitor add-on treatment was the primary endpoint and tested by Student's *t*-test, Wilcoxon signed rank test, or analysis of variance (ANOVA). The change in HbA1c was examined as the dependent variable and tested against duration of diabetes, gender, body mass index (BMI), age, ethnicity, and baseline HbA1c as independent variables by multiple regression analysis. The change in HbA1c was also studied by analysis of covariance adjusted for baseline HbA1c, duration of diabetes, BMI, and ethnicity. Categorical variables were compared by *χ* ^2^ test. Statistical significance was based on 2-sided tests and accepted at the *P* < 0.05 level.

## 3. Results

Amongst the 80 patients studied, 64 were taking Sitagliptin, 12 were on Saxagliptin, and 4 were on Vildagliptin. The demographic and clinical characteristics of these patients are shown in Table 1. After triple oral therapy duration of 4.6 (3.6–6.6) months, their mean HbA1c decreased from 8.3% (7.7–8.9) [67 mmol/mol, 61–74] to 7.2% (6.8–7.6%) [55 mmol/mol, 51–60 mmol/mol] with 26 patients (32.5%) achieving an HbA1c of <7% (53 mmol/mol). Weight before (79.6 ± 19.5 kg) and after (78.8 ± 19.3 kg) triple therapy did not change significantly in the 66 patients with readings available before and after this treatment.

The mean changes in HbA1c from pre- to posttriple therapy, according to clinical characteristics, are shown in Table 2. Patients with higher baseline HbA1c, absence of obesity, and younger age showed a greater response. By multiple regression analysis, these three factors collectively account for 32% of the variance in HbA1c response. Gender, the duration of diabetes, and BMI analyzed as a continuous variable were not significant factors. The duration of triple therapy analyzed categorically (≥ or <6 months) or by regression did not affect response (*r* = −0.17).

There were 41 Asian and 39 non-Asian patients in this study. Their results are summarized in Table 3. The Asian and non-Asian groups have similar age, duration of diabetes, and baseline glycaemic control. The BMI of the Asian group was 5.0 kg/m^2^ lower. The HbA1c reduction after the implementation of triple therapy was not different between the Asian (1.00, 0.6–1.3%) and non-Asian group (0.90, 0.4–1.6%). Analysis of covariance showed that ethnicity was not a significant determinant of HbA1c response.

## 4. Discussion

Metformin and sulphonylurea are commonly prescribed together in patients with type 2 diabetes [1, 2]. When such combination therapy can no longer maintain acceptable glycaemic control, many other antihyperglycaemic agents are available to be added as the third agent of triple therapy. McIntosh et al. conducted a systematic review and meta-analysis of 33 controlled trials to evaluate the comparative safety and efficacy of various classes of antihyperglycaemic therapies in this scenario [15]. Insulins, DPP-4 inhibitor, glucagon-like peptide-1 (GLP-1) analogues, and thiazolidinediones (TZDs) all produced statistically significant reductions in HbA1c ranging from −0.89% to −1.17%, whereas meglitinides and alpha-glucosidase inhibitors did not. In their analysis, insulins and TZDs were associated with weight gain of 1.85–5.00 kg, GLP-1 analogues were associated with modest weight loss, and DPP-4 inhibitor was weight-neutral and did not show any increase in hypoglycaemia which was found when insulin was used. In the analysis of McIntosh, only one DPP-4 inhibitor (Sitagliptin) was included and when used in triple therapy, a fall in HbA1c of 0.89% was demonstrated [12]. In another study not included in the analysis, Linagliptin administrated for a 24-week period as triple therapy significantly improved glycaemic control (HbA1c, 0.62%) and was well tolerated [13]. Moreover, Chien et al. study showed that Sitagliptin as the 4th agent also reduced HbA1c by about 1% when added to the combination of metformin, sulphonylurea, and glucosidase-inhibitor [16]. Based on these studies, DPP-4 inhibitorsas a third-line oral antihyperglycaemic agent appear to be effective and safe, without weight gain. Overall, despite its increasing usage, there have been few reports on the efficacy of DPP-4 inhibitors as the third oral agent in a clinical setting.

Also of considerable interest, Kim et al. in their review of DPP-4 inhibitors provided evidence that this class of agents may be more effective in Asian patients [14]. However, the great majority of the studies included in the analysis pertained to mono- or dual therapies. The categorization of ethnicity was not based on an individual patient basis. Instead, it assigned the total cohort of a trial as Asian or non-Asian ethnicity according to whether the study included more or less than 50% Asian participants or was conducted in an Asian dominant country. The superiority of any pharmacological agent in an Asian population could have important medicoeconomic implications due to the high and progressively increasing prevalence of diabetes in Asia. It could also provide insight to a differing pathogenesis of diabetes in Asians.

Based on these considerations we have audited the efficacy of DPP-4 inhibitors as the third oral agent in our clinic and also made comparison of the response in Asian and non-Asian patients. Our results confirmed the usefulness of DPP-4 inhibitors in improving glycaemic control in this context. One-third of patients reached an optimal level of glycaemic control, as defined by an HbA1c of less than 7% (53 mmol/mol). The efficacy of DPP-4 inhibitors in this clinical setting seems quite effective although we could not dissect out the impact of a concerted effort at lifestyle changes by the patients when faced with the possibility of insulin requirement. Baseline glycaemic control, degree of obesity, and age of patients were identified by both univariate and multivariate analyses to be important determinants of response. Not unexpectedly, the drop in HbA1c was greater in those with worse initial glycaemic control. There was also a better response in those with lesser obesity, perhaps a reflection that these subjects are relatively more insulin deficient than insulin resistant in the pathogenesis of their diabetes. They would be expected to respond better to an agent that can increase insulin availability. Our observation that the younger patients appeared to respond better is a potential advantage of the DDP-4 inhibitors as the need for better glycaemic control is obviously more in this age group. It is interesting that duration of diabetes does not seem to impair response, suggesting that the ability of DPP-4 inhibitors to augment meal associated insulin secretion does not decline with time of having diabetes. Moreover, there was no evidence of a significant decline in its efficacy up to 12 months of such triple therapy. However, the benefits of such treatment regimen for the longer term would need to be examined.

The findings of Seino et al. suggest a better response of Asian patients to DPP-4 inhibitors, raising the possibility that patients of Asian ethnicity have relatively more defects in meal associated insulin secretion [17, 18]. A better response to glucagon-like peptide analogues in Asians has also been shown in a meta-analysis [19]. Our study did not provide evidence to support this notion of difference in ethnic response. In comparison with the reviews and meta-analyses mentioned above, our study has the advantage of categorizing ethnicity specifically at an individual level but had examined only 80 subjects. Therefore, we cannot exclude the possibility that a small difference may be missed. Our study also only examined patients on triple oral agent therapy which was not comprehensively evaluated in the Kim analysis. So the possibility remains that Asian patients would respond better to DPP-4 inhibitors in mono- or dual therapies. Being a retrospective study, we cannot be certain that there was no selection bias for using DPP-4 inhibitor as the third agent but, during the period examined, the safety of the thiazolidinediones had come into question and the SGLT-2 inhibitors were not yet available. The DPP-4 inhibitors were effectively the only third oral agent available to us.

It would have been informative to know the relative efficacy of the DPP-4 inhibitor in comparison with insulin or GLP-agonist in this clinical scenario. However, this would require a randomized clinical trial and is beyond the scope of this analysis. However, our study confirmed the effectiveness of DPP-4 inhibitors as the third oral agent in improving glycaemic control in type 2 diabetes.

## Figures and Tables

**Table 1:** Demographic and clinical characteristic of participants at baseline.

Characteristics	Total
	*n* = 80
Gender (*n*)	
Male/female	51/29
Ethnicity (*n*)	
Asians/non-Asians	41/39
Age (years)	62.0 ± 9.3
Diabetes duration (years)	12.7 ± 5.8
Weight (kg)	77.6 (68.8–93.1)
Body mass index (kg/m^2^)	27.5 (25.2–31.8)
HbA1c (%)	8.3 (7.7–8.9)
HbA1c (mmol/mol)	67 (61–74)

**Table 2:** Changes in HbA1c: pre- versus posttriple therapy.

Subgroups	Mean change in HbA1c from baseline (%)	Test statistics
Baseline HbA1c		
<8% (*n* = 32)	−0.64	*F* = 21.4; *P* = 0.000
8-9% (*n* = 35)	−1.12	
>9% (*n* = 13)	−2.10	
Obesity∗		
No (*n* = 46)	−1.18	*Z* = −2.0; *P* = 0.04
Yes (*n* = 34)	−0.96	
Age		
<65 years (*n* = 49)	−1.25	*t* = 2.3; *P* = 0.02
≥65 years (*n* = 31)	−0.82	
Gender		
Male (*n* = 51)	−1.05	*Z* = 0.4; *P* = 0.7
Female (*n* = 29)	−1.15	
Diabetes duration		
<10 years (*n* = 29)	−1.18	*F* = 0.3; *P* = 0.46
10–20 years (*n* = 42)	−1.00	
>20 years (*n* = 9)	−1.20	
Duration on triple therapy		
<6 months (*n* = 56)	−1.19	*t* = 1.7; *P* = 0.1
≥6 months (*n* = 24)	−0.85	

∗Obese in Asians: BMI ≥ 28 kg/m^2^; obese in non-Asians: BMI ≥ 30 kg/m^2^.

**Table 3:** Demographic characteristics and clinical response: Asian versus non-Asian groups.

	Asian	Non-Asian	Test statistics
	*n* = 41	*n* = 39	
Age (years)	61.6 ± 10.3	62.3 ± 8.3	*t* = 0.3; *P* = 0.8
Weight (kg)	69.0 (60.2–77.6)	89.0 (77.5–100.1)	*Z* = 5.2; *P* = 0.000
Body mass index (kg/m^2^)	25.6 (24.0–27.5)	31.6 (28.2–34.4)	*Z* = 5.4; *P* = 0.000
Diabetes duration (years)	13.7 ± 5.8	11.7 ± 5.7	*t* = −1.6; *P* = 0.1
Duration on triple therapy (months)	4.6 (3.7–6.4)	4.5 (3.5–7.2)	*Z* = −0.1; *P* = 0.9
Baseline HbA1c			
(%)	8.1 (7.6–8.7)	8.3 (7.8–8.9)	*Z* = 1.6; *P* = 0.9
(mmol/mol)	65 (60–72)	67 (62–74)	
Change in weight from baseline (kg)	−0.7 ± 1.6	−1.0 ± 2.4	*t* = −0.6; *P* = 0.6
Change in HbA1c from baseline (%)	−1.00 (0.6–1.3)	−0.90 (0.4–1.6)	*Z* = 0.0; *P* = 1.0
Patients achieving a given HbA1c			
<7% (53 mmol/mol)	39.0	25.6	*X* ^2^ = 1.6; *P* = 0.2
<8% (64 mmol/mol)	90.2	76.9	*X* ^2^ = 2.6; *P* = 0.1
